# Supplementary material for: Novel Pseudomonas Species Prevent the Growth of the Phytopathogenic Fungus Aspergillus flavus
Source: BioTech (Basel). 2024 Mar 30;13(2):8. doi: 10.3390/biotech13020008 (PMC11036216; doi:10.3390/biotech13020008)
Supplement: Supplementary file 1 [file biotech-13-00008-s001.zip › Supplementar material.pdf]

Novel *Pseudomonas* species prevent the growth of the phytopathogenic fungus *Aspergillus flavus*

Franciene Rabiço Oliveira[a,b], Tiago Cabral Borelli[a,c], Maria de Lourdes Teixeira de Moraes Polizeli[b], Ricardo Roberto da Silva[c], Rafael Silva-Rocha[d], María-Eugenia Guazzaroni[b]*

[a]Department of Cell and Molecular Biology, Faculdade de Medicina de Ribeirão Preto, University of São Paulo, São Paulo, SP, Brazil

[b]Department of Biology, Faculdade de Filosofia, Ciências e Letras de Ribeirão Preto, University of São Paulo, São Paulo, SP, Brazil

[c]Department of Biomolecular Sciences, Faculdade de Ciências Farmacêuticas de Ribeirão Preto, University of São Paulo, Ribeirão Preto, Brasil

[d]ByMyCell Inova Simples. Av. Dra. Nadir Aguiar, 1805 – Supera Parque, Ribeirão Preto, SP, Brazil

*Correspondence to: María-Eugenia Guazzaroni, meguazzaroni@ffclrp.usp.br

Faculdade de Filosofia, Ciências e Letras de Ribeirão Preto, Universidade de São Paulo.

Av. Bandeirantes, 3.900. CEP: 14049-901, Ribeirão Preto, São Paulo, Brazil.

Supporting Materials

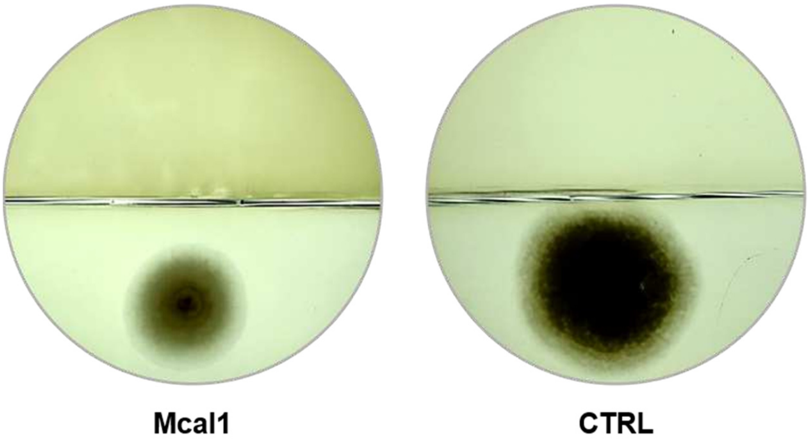

Figure S1. Antibacterial activity of VOCs of Mcal1 strain on LB medium in 2-compartment Petri dishes.

Table S1. Digital DNA-DNA hybridization (dDDH) values between the BJa3 genome and the selected typestrain genomes.

Strain	dDDH (d4, in %)
Pseudomonas soli LMG 27941	92,1
Pseudomonas maumuensis COW77	56,5
Pseudomonas peradeniyensis BW13M1	42,1
Pseudomonas mosselii DSM 17497	40,9
Pseudomonas muyukensis COW39	36,4
Pseudomonas xantholysinigenes RW9S1A	35,5
Pseudomonas sichuanensis WCHPs060039	34,5
Pseudomonas oryziphila 1257T	34,2
Pseudomonas entomophila L48	34,1
Pseudomonas xanthosomatis COR54	32,6
Pseudomonas fakonensis COW40	32,4
Pseudomonas wayambapalatensis RW3S1	28,8
Pseudomonas taiwanensis DSM 21245	28,1

Table S2. Digital DNA-DNA hybridization (dDDH) values between the MCal1 genome and the selected typestrain genomes.

Strain	dDDH (d4, in %)
Pseudomonas glycinae MS586	52
Pseudomonas gozinkensis LMG 31526	51,6
Pseudomonas kribbensis KCTC 32541T	42,9
Pseudomonas allokribbensis LMG31525T	42,5
Pseudomonas bananamidigenes BW11P2	39,4
Pseudomonas botevensis COW3	34,6
Pseudomonas koreensis LMG 21318	34,2
Pseudomonas koreensis JCM 14769	34,1
Pseudomonas atagonensis PS14	33,6
Pseudomonas monsensis PGSB 8459	33,2
Pseudomonas ekonensis COR58	33
Pseudomonas iranensis SWRI54	31,8
Pseudomonas atacamensis M7D1	31,7
Pseudomonas moraviensis LMG 24280	31,6

Table S3. Genome statistics for *Pseudomonas sp.* BJa3 and MCal1.

Attribute	BJa3	MCal1
Size (bp)	5952323	6264134
contigs (nº)	750	155
coverage (Avg)	270	251.1
GC (%)	64.05	60.42
Coding sequences	5272	5650
tRNA	4	73
rRNA	77	5
tmRNA	1	1
Plasmid	0	0
GenBank	JAOXMC000000000	JAKUMP000000000
SRA	PRJNA808800	PRJNA808800
Isolated from	Garden soil	Sugarcane Juice
